# Pulmonary edema ex vacuo after drainage of pyo-pneumothorax

**DOI:** 10.1186/s12890-023-02739-3

**Published:** 2023-11-16

**Authors:** Nahid Zaghba, Zakaria Laklaai, Khadija Chaanoun, Hanane Benjelloune, Najiba Yassine

**Affiliations:** Department of Respiratory Diseases Ibn Rochd Casablanca, Casablanca, Morocco

**Keywords:** Pulmonary edema ex vacuo, Re-expansion pulmonary edema, Hydro-pneumothorax, Pleural drainage, Mycobacterium tuberculosis

## Abstract

This case presents a rare occurrence of re-expansion pulmonary edema following a drainage of pyo-pneumothorax in a 33-year-old patient. The diagnosis was established through a thoracic radiography, and the treatment consisted of symptomatic management, showing positive progress. Later on, the patient was diagnosed with pleural tuberculosis via GeneXpert testing and subsequently initiated on anti-bacterial therapy.

This case report aims to shed light on the infrequent pulmonary edema ex vacuo as a complication of pleural drainage. It explores its causes, risk factors, diagnostic approaches, and treatment options. this study highlights the necessity of effective prevention and management strategies.

## Case presentation

We present the case of a 33-year-old male patient with a smoking history of 20 pack-years, without any significant medical history. He presented with dyspnea, classified as stage 2 on the Modified Medical Research Council (mMRC) scale since 1 month, along with weight loss. He reported a recent worsening of symptoms over the past week, accompanied by right thoracic pain. Initial assessment revealed an oxygen saturation of 89% on ambient air, a normal pulse rate of 88 beats per minute, and a blood pressure of 130/84 mmHg. Additionally, the patient had a fever, with a body temperature of 38.6°C and BMI of 16,5.

**Fig. 1:**
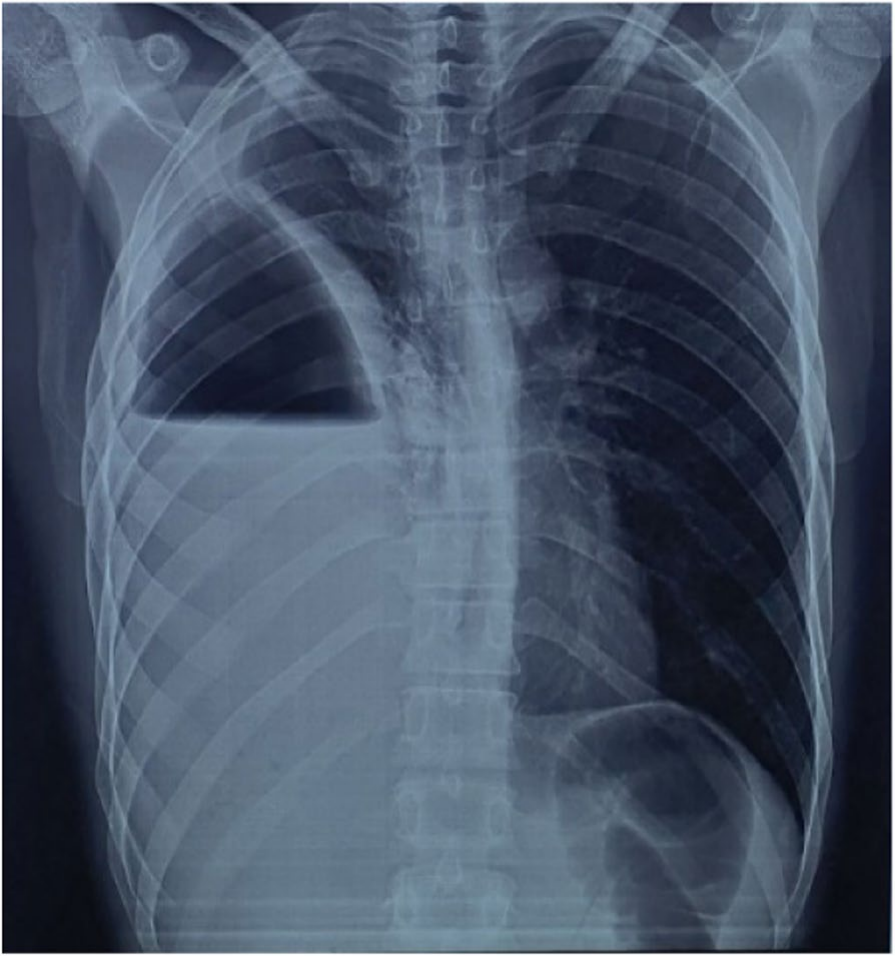
Chest x ray at admission showing a right hydro-pneumothorax

A chest X-ray was performed, which showed a hydro-pneumothorax on the right side, while the left hemithorax appeared normal (Fig. 1).

To treat the hydro-pneumothorax, an intercostal tube was placed in the 5th intercostal space, along the mid-axillary line on the right side. A total of 1 L of purulent fluid were successfully drained. However, within the following hour, the patient's dyspnea worsened, with an oxygen saturation of 80%, hypotension (blood pressure of 8/5 mmHg), and tachycardia (heart rate of 113 beats per minute). Crepitants were detected at auscultation. As a result, the patient was immediately admitted to the intensive care unit for close monitoring.

**Fig. 2:**
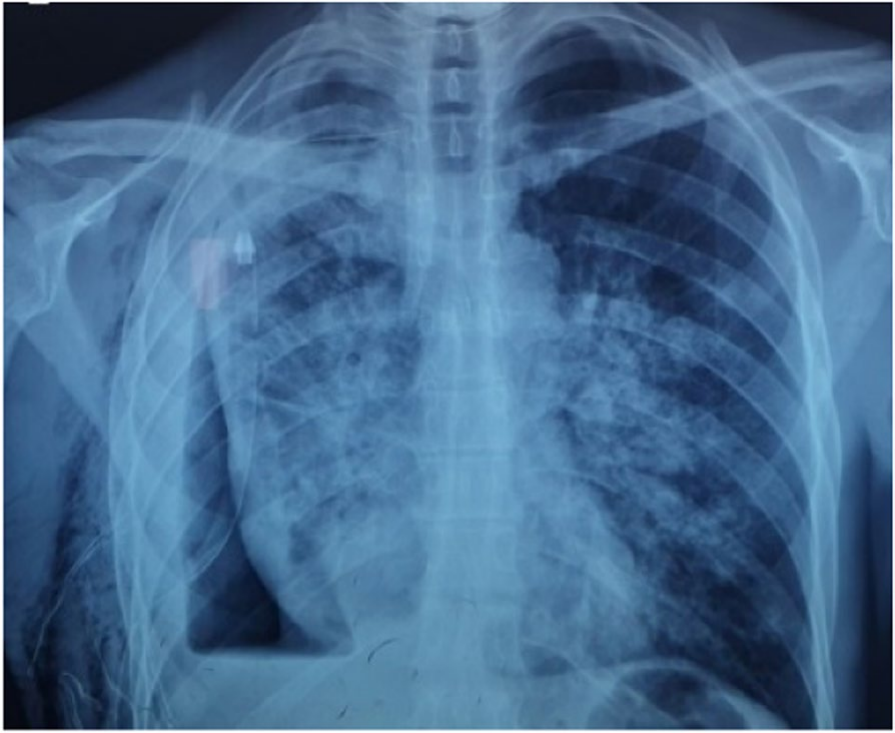
Chest X-ray 3h after drainage reveals a bilateral infiltrative pattern, substantial regression of the pyo-pneumothorax and subcutaneous emphysema

An subsequent chest X-ray was performed, which revealed the development of a bilateral infiltrative pattern and regression of the pyo-pneumothorax, consistent with re-expansion pulmonary edema, as well as subcutaneous emphysema (Fig. 2).

**Fig. 3:**
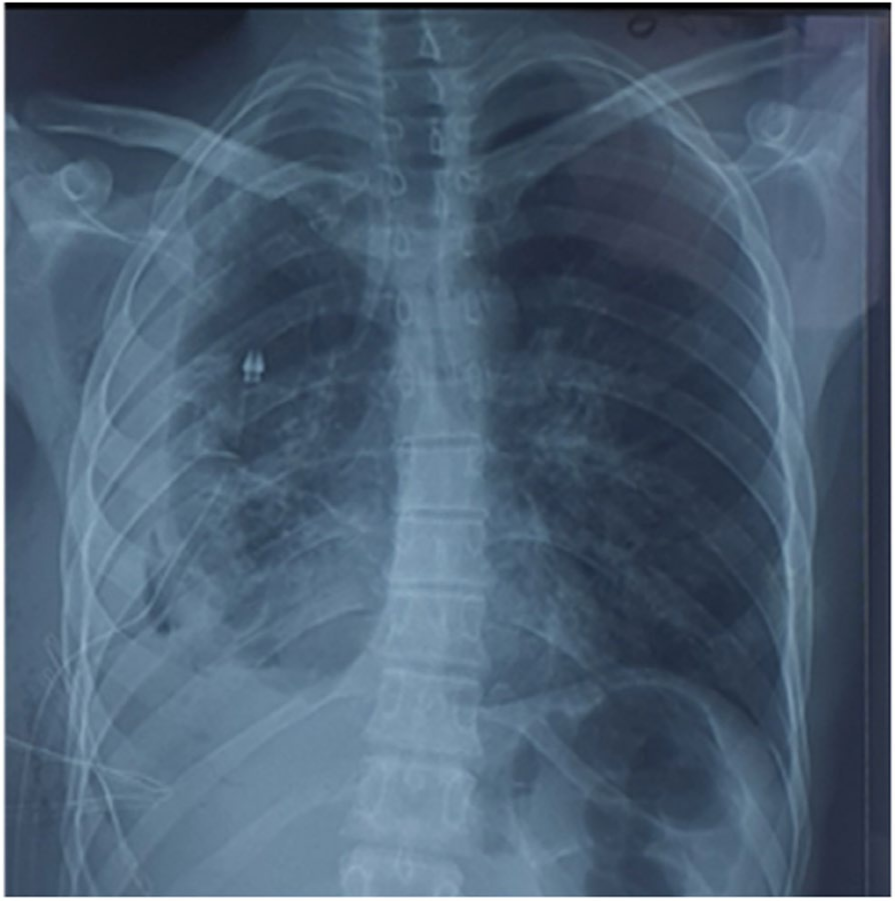
Resolution of the pulmonary edema 24 h later

The primary treatment approach was focused on symptomatic measures. Oxygen therapy was administered to improve oxygenation, and the drainage tube was clamped to prevent further fluid loss. Over the course of 48 to 72 h, the patient's condition showed favorable evolution with the resolution of symptoms clinically and radiologically (Fig. 3).

**Fig. 4:**
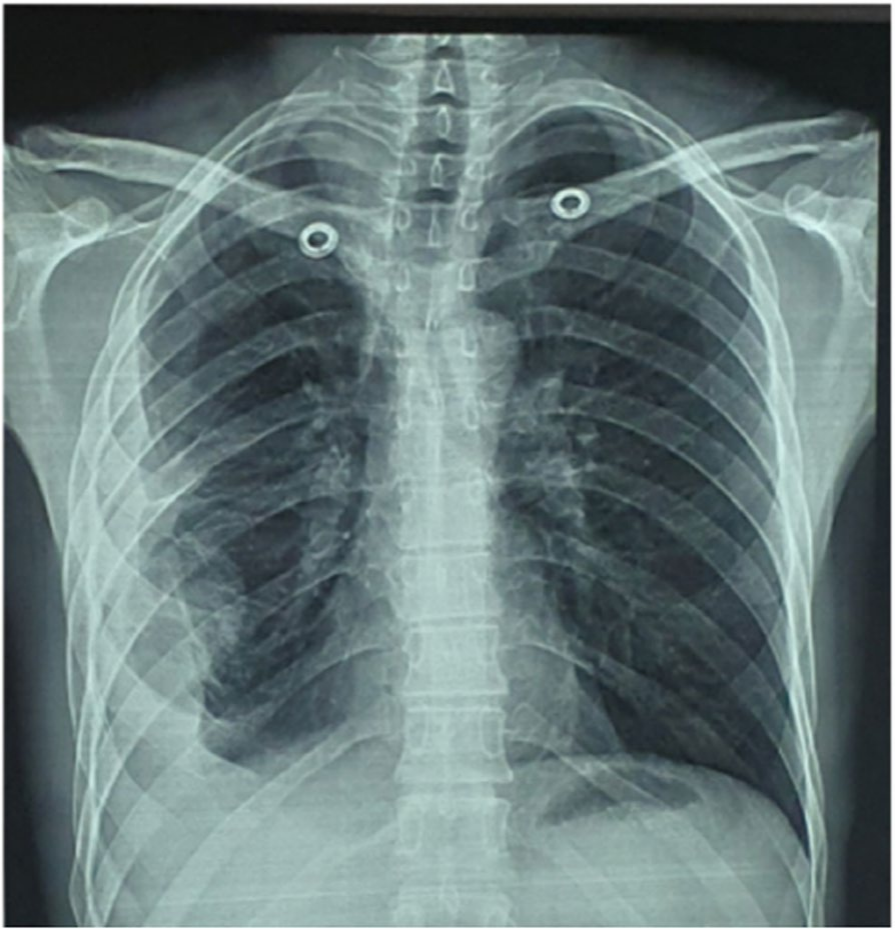
Chest x ray after 6 month showing pachypleurite

The etiology of the pyo-pneumothorax was established through GeneXpert analysis of the pleural fluid, which detected the presence of *Mycobacterium tuberculosis*. Following this diagnostic, we initiate an anti-bacillary treatment, leading to notable enhancements in both clinical symptoms and radiological findings (Fig. 4).

## Discussion

Pulmonary edema ex vacuo is a relatively uncommon complication that can arise as a result of pleural drainage procedures. The earliest documentation of this condition dates back to 1853 when Pinault first described it. Nearly a century later, in 1959, Carlson et al. published case report detailing Re-expansion pulmonary edema (REPE) development subsequent to the drainage of a pneumothorax. The diagnosis is typically established through radiological imaging and clinical finding. While no specific curative treatment exists, Patient’s management primarily revolves around alleviating symptoms and close monitoring. The mortality rate associated with REPE varies between 5 to 10% [1].

The pathophysiology is complex and influenced by various factors, including prolonged collapse of the lungs, changes in capillary permeability, and heightened hydrostatic pressure. The alterations in capillary permeability are further exacerbated by hypoxemia, damage to capillary walls, and the presence of inflammatory mediators. These factors lead to the overflow of fluid and proteins into the interstitial space and alveoli [2]. The study by Feller et al. [3] reported an increase in the incidence from 0.5% to 2.7% after performing follow-up radiographs suggesting that the true incidence of REPE may be underestimated, Various risk factors have been suggested, such as prolonged collapse, larger pneumothorax size, younger age, and underlying medical conditions knowing these risk factors is crucial for effective prevention strategies. As for our case, the risk factors are prolonged collapse duration and the size [2]. The presentation can vary widely, showing a broad array of symptoms. Patients might have no symptoms in some cases, while in others, it may manifest as respiratory distress. this condition can imitate cardiogenic pulmonary edema, making diagnosis complex [4]. chest radiographs assess the diagnostic for pulmonary edema characterized by interstitial opacity, consolidations, air bronchogram, as well as distinct lung clefts [3]. Usualy unilateral, and bilateral are rare like our case,the underlying causes for this presentation (bilateral) are believed to involve mediastinal deviation, systemic cytokine activity, and heightened cardiac output [5]. In a study conducted by Kim et al., Performing thoracic scans on all patients led to a significant improvement in the incidence rate of REPE, increasing it from 19% to 29.8%. the CT scans are more sensitive than standard radiography in diagnosing REPE, frequent finding is ground glass opacity, septal thickening and alveolar consolidations [6], The progression of reexpansion pulmonary edema can vary greatly, ranging from spontaneous resolution to potentially fatal respiratory failure however resolution generally occurs between 24 and 72 h, and radiological normalization can be observed on the 5th to 7th day. A review study assessing mortality rates reported 11 death out of 53 (21%) [7]. No specific recommendation exists at present for this pathology. oxygen therapy is crucial in cases of hypoxia. Some authors propose the use of non-invasive ventilation with positive end-expiratory pressure (PEEP) as a possible approach. Additionally stopping pleural aspiration by clamping the drain is an important part of the treatment [8]. The British Thoracic Society 2010 guidelines recommend avoiding high intrapleural pressures during the procedures. It also emphasizes that a maximum of 1.5 L should be drained in the first hour after insertion of the drain [9]. One effective approach to prevent respiratory distress during the drainage of pleural cavities is to closely monitor the pleural pressure and ensure it remains within a safe range of up to -20 cmH2O. By carefully managing the pressure, larger volumes of fluid, approximately up to 5,000 ml per thoracentesis, can be removed. The occurrence of chest discomfort during thoracentesis in an indicator of potentially decreases in pleural pressure (Ppl) values, signaling the need to halt the procedure. However, the development of a cough alone does not necessarily require the end of thoracentesis [10]. As for pleural tuberculosis diagnosis challenges continue due to low pleural fluid test sensitivity. Biomarkers like ADA and PCR help, while pleural biopsy remains the gold standard. For our patient, diagnosis was achieved by Gene Xpert analysis of pleural fluid, where sensitivity is higher in empyema cases. Italian study (419 participants) noted more internal mammary lymph nodes in tuberculous pleurisy (77.2%), aiding pre-test probability assessment [11].

## Conclusion

This case highlights the rare occurrence of re-expansion pulmonary edema following pleural drainage. early recognition, appropriate management are crucial and preventive measures for pleural fluid drainage can minimize its occurrence. Ongoing research will further enhance our approach to managing this complication, ensuring better outcomes for patients undergoing pleural interventions.

## Data Availability

All data and materials are available for sharing, if needed, by the corresponding author.

## References

[1] Ault MJ, Rosen BT, Scher J, Feinglass J, Barsuk JH. Thoracentesis outcomes: a 12-year experience. Thorax. 2015;70(2):127–132. 10.1136/thoraxjnl-2014-206114 10.1136/thoraxjnl-2014-206114 25378543

[2] Eduardo Henrique Genofre, Francisco S. Vargas and Lisete R. Teixeira et al. Reexpansion pulmonary edema. J. Pneumologia*.* 29(2):101–106. 10.1590/S0102-35862003000200010

[3] Feller kopman d, walkey a, berkowitz d. the relation ship of pleural pressure to symptom development during therapeutic thoracentesis. chest 2006. 129: 1556–60. 10.1378/chest.129.6.1556 16778274

[4] Benhamed A, Tazarourte K. Bilateral re-expansion pulmonary edema: an uncommon complication of the pneumothorax drainage. Arch Clin Cases. 2021;7(1):10–14. 10.22551/2020.26.0701.10166. PMID: 34754921; PMCID: PMC8565686. 10.22551/2020.26.0701.10166 PMC8565686 34754921

[5] Haga T, Kurihara M, Kataoka H. Risk for re-expansion pulmonary edema following spontaneous pneumothorax. Surg Today. 2014;44(10):1823–1827. 10.1007/s00595-013-0726-y 10.1007/s00595-013-0726-y 24065192

[6] Kim YK, Kim H, Lee CC, Choi HJ, Lee KH, Hwang SO (2009). new classification and clinical characteristics of reexpansion pulmonary edema after treatment of spontaneous pneumothorax. Am J Emerg Med.

[7] Mahfood S, Hix WR, Aaron BL, Blaes P, Watson DC (1988). Reexpansion pulmonary edema. Ann Thorac Surg.

[8] Papakonstantinou DK, Gatzioufas ZI, Tzegas GI (2007). Unilateral pulmonary oedema due to lung re-expansion following pleurocentesis for spontaneous pneumothorax. The role of non-invasive continuous positive airway pressure ventilation. Int J Cardiol.

[9] Roberts ME, Neville E, Berrisford RG, Antunes G, Ali NJ; BTS Pleural Disease Guideline Group. Management of a malignant pleural effusion: British Thoracic Society Pleural Disease Guideline 2010. Thorax. 2010;65 Suppl 2:ii32-ii40. 10.1136/thx.2010.136994 10.1136/thx.2010.136994 20696691

[10] Feller-Kopman D, Walkey A, Berkowitz D, Ernst A. The relationship of pleural pressure to symptom development during therapeutic thoracentesis. Chest. 2006;129(6):1556–1560. 10.1378/chest.129.6.1556. 10.1378/chest.129.6.1556 16778274

[11] Levi G, Rocchetti C, Mei F, et al. Diagnostic role of internal mammary lymph node involvement in tuberculous pleurisy: a multicenter study [published online ahead of print, 2022 Feb 18]. Pulmonology. 2022;S2531–0437(22)00022–8. 10.1016/j.pulmoe.2022.01.010 10.1016/j.pulmoe.2022.01.010 35190300

